# Electrochemical Co-Reduction Synthesis of AuPt Bimetallic Nanoparticles-Graphene Nanocomposites for Selective Detection of Dopamine in the Presence of Ascorbic Acid and Uric Acid

**DOI:** 10.3390/s150716614

**Published:** 2015-07-09

**Authors:** Zongya Zhao, Mingming Zhang, Xiang Chen, Youjun Li, Jue Wang

**Affiliations:** 1 The Key Laboratory of Biomedical Information Engineering of Ministry of Education, Institute of Biomedical Engineering, School of Life Science and Technology, Xi’an Jiaotong University, Xi’an 710049, China; E-Mails: zhaozongya2010@stu.xjtu.edu.cn (Z.Z.); zhangmingming@stu.xjtu.edu.cn (M.Z.); chenxiang@mail.xjtu.edu.cn (X.C.); liyoujun122@stu.xjtu.edu.cn (Y.L.); 2 National Engineering Research Center of Health Care and Medical Devices, Xi’an Jiaotong University Branch, Xi’an 710049, China

**Keywords:** AuPt bimetallic nanoparticles-graphene nanocomposite, electrochemical co-reduction, dopamine

## Abstract

In this paper, AuPt bimetallic nanoparticles-graphene nanocomposites were obtained by electrochemical co-reduction of graphene oxide (GO), HAuCl_4_ and H_2_PtCl_6_. The as-prepared AuPt bimetallic nanoparticles-graphene nanocomposites were characterized by scanning electron microscopy (SEM), electrochemical impedance spectroscopy (EIS) and other electrochemical methods. The morphology and composition of the nanocomposite could be easily controlled by adjusting the HAuCl_4_/H_2_PtCl_6_ concentration ratio. The electrochemical experiments showed that when the concentration ratio of HAuCl_4_/H_2_PtCl_6_ was 1:1, the obtained AuPt bimetallic nanoparticles-graphene nanocomposite (denoted as Au1Pt1NPs-GR) possessed the highest electrocatalytic activity toward dopamine (DA). As such, Au1Pt1NPs-GR nanocomposites were used to detect DA in the presence of ascorbic acid (AA) and uric acid (UA) using the differential pulse voltammetry (DPV) technique and on the modified electrode, there were three separate DPV oxidation peaks with the peak potential separations of 177 mV, 130 mV and 307 mV for DA and AA, DA and UA, AA and UA, respectively. The linear range of the constructed DA sensor was from 1.6 μM to 39.7 μM with a detection limit of 0.1 μM (*S/N* = 3). The obtained DA sensor with good stability, high reproducibility and excellent selectivity made it possible to detect DA in human urine samples.

## 1. Introduction

In recent years, bimetallic nanoparticles have gained significant research attention and have been widely applied in a variety of fields, such as electrochemical sensors [1,2,3,4,5,6,7], fuel cells [8,9,10,11,12], and raman scattering [13] due to their unique physical and chemical properties. Compared with the corresponding monometallic nanoparticles, bimetallic nanoparticles possessed many favorable characteristics including increased surface area, improved electron transfer rate, enhanced electrocatalytic activity and promoted stability, which mainly resulted from the synergistic effects of these two monometallic nanoparticles. Graphene (GR), a popular material in the field of materials science, has attracted the interest of the scientific community because of its extremely high surface area, good electroconductivity, and physical strength. Bimetallic nanoparticles-GR nanocomposites, which showed excellent electrochemical properties because of the synergistic effects of bimetallic nanoparticles and GR, have recently been extensively studied and applied to fabricate various electrochemical sensors, such as erythromycin sensors [14], dopamine sensors [15], glucose sensors [16], nitric oxide sensors [17], 4-nitrophenol sensors [18], and nitrite sensors [19,20].

Compared with chemical preparation methods, electrochemical synthesis of bimetallic nanoparticles, which has recently gained significant research attention, shows lots of advantages, such as simple and fast synthesis, not using highly toxic reducing agents, and easily controlling the shape, size, composition and density of bimetallic nanoparticles by changing deposition potential, time, and concentration of metal precursor solutions [3,21]. Recently, the electrochemical method has been reported to prepare GR from graphene oxide (GO) by applying a constant cathodic potential [22]. At such a negative potential, metal precursors such as HAuCl_4_ or H_2_PtCl_6_ can be easily electrochemically reduced to Au nanoparticles or Pt nanoparticles. For example, Liu *et al.* prepared GR-three dimensional nanostructure Au nanocomposite by immersing graphene oxide modified electrode into HAuCl_4_ precursor solution and a one-step electrochemical co-reduction was subsequently performed by applying a constant cathodic potential [23]. Fu *et al.* presented an approach whereby GO and HAuCl_4_ were electrochemically co-reduced in ionic liquid to prepare GR-Au nanocomposites [24]. Zhou *et al.* proposed a one-step electrochemical approach to the synthesis of highly dispersed Pt nanoparticles on GR [25]. As such, it is possible to prepare bimetallic nanoparticles-GR nanocomposite by electrochemical co-reduction methods at an appropriate cathodic potential. In this work, cauliflower-like AuPt bimetallic nanoparticles-GR nanocomposites were obtained by electrochemical co-reduction of GO, HAuCl_4_ and H_2_PtCl_6_.

Dopamine (DA), one of the most significant neurotransmitters, can greatly affect the role of human metabolic, brain nervous, cardiovascular and hormonal systems [26,27]. Irregular concentration levels of DA may lead to central nervous diseases such as primary Parkinsonism and schizophrenia [28]. It is well-known that direct electrochemical detection of DA at bare electrodes is nearly impossible, because interfering substances such as ascorbic acid (AA) and uric acid (UA) usually co-exist with DA in body fluids outside of cells at a high concentration level and can be oxidized within the same potential range as that of DA. Therefore, to address these problems, many composites, such as ion-exchange membranes [29], conducting polymer films [30,31], carbon nanotubes [32,33,34], metal and metal oxide nanoparticles [35,36,37,38,39,40], tyrosinase-based materials [41,42], have been used to enhance the selectivity and sensitivity of electrodes. However, to the best of our knowledge, there is no report about the use of cauliflower-like AuPt bimetallic nanoparticles-GR nanocomposites prepared by electrochemical co-reduction of GO, HAuCl_4_ and H_2_PtCl_6_ for selective detection of DA in the presence of AA and UA.

In this paper, cauliflower-like AuPt bimetallic nanoparticles-GR nanocomposites were prepared directly on electrodes via electrochemical co-reduction methods. Generally, GO suspension was first dropped onto the surface of electrodes to get GO modified electrodes. Next, GO modified electrodes were immersed into a precursor solution containing HAuCl_4_ and H_2_PtCl_6_ and a one-step electrochemical co-reduction was subsequently performed by applying a constant cathodic potential. The obtained nanocomposites were characterized using electrochemical methods in detail and results indicated that when the concentration ratio of HAuCl_4_/H_2_PtCl_6_ was 1:1, the AuPt bimetallic nanoparticles-graphene nanocomposite (denoted as Au1Pt1NPs-GR) possessed the highest electrocatalytic activity toward DA. As such, Au1Pt1NPs-GR nanocomposite was used to detect DA in the presence of AA and uric acid UA using the differential pulse voltammetry (DPV) technique. The obtained DA sensor showed good selectivity and sensitivity toward DA, and the results of dopamine detection in human urine samples was satisfactory.

## 2. Experiment

### 2.1. Materials and Reagents

Graphite oxide (purity, >99%) was obtained from Xfnano Materials Tech. Co., Ltd. (Nanjing, China). Chloroauric acid tetrahydrate (HAuCl_4_·4H_2_O) and chloroplatinic acid hexahydrate (H_2_PtCl_6_·6H_2_O) was purchased from Sangon Biotech. Co., Ltd. (Shanghai, China) and Aladdin Industrial Inc. (Shanghai, China), respectively. AA, UA and DA were obtained from Sinopharm Chemical Reagent Co., Ltd. (Shanghai, China). All other reagents were directly used without further purification. All aqueous solutions were prepared using ultrapure water (Millipore, ≥18 MΩcm, Billerica, MA, USA). The phosphate buffer solution (PBS, 0.1 mol/L) was prepared using 0.1 mol/L Na_2_HPO_4_ and 0.1 mol/L NaH_2_PO_4_ and the pH value of PBS was adjusted by mixing the stock solutions of NaH_2_PO_4_ and Na_2_HPO_4_ at different rations.

### 2.2. Equipment

All electrochemical measurements were performed on a CHI (Chenhua Instrument company, Shanghai, China) 650E electrochemical workstation with a three-electrode system consisting of a glassy carbon electrode (diameter: 3 mm), an Ag/AgCl (with 3 M KCl) reference electrode and a platinum wire auxiliary electrode. 0.1 M PBS was used as an electrolyte solution. Cyclic voltammetric experiments were performed with a scan rate of 100 mV/s. All experiments were carried out at room temperature. Scanning electron microscopic images were collected on a Jsm-7800f field emission scanning electron microscopy (Electro Co., Tokyo, Japan). X-ray diffraction (XRD) patterns were carried out on an X’PertPro PANalytical diffractometer using Cu Kα radiation (λ = 0.15418 nm).

### 2.3. Synthesis of AuPt Bimetallic Nanoparticles-GR Nanocomposites

Prior to preparation, glassy carbon electrode (GCE) was polished successively with 1.0, 0.3 and 0.05 μm alumina powder to form a mirror surface on a polishing cloth and rinsed thoroughly with ultrapure water between each polishing step. Then, the GCE was sonicated in ethanol (1:1), HNO_3_ (1:1) and ultrapure water in sequence, and finally dried in air. The purchased graphite oxide was dispersed into ultrapure water by ultrasonication for 2 h to obtain homogeneous GO dispersion with a concentration of 0.5 mg/mL. Subsequently, 7 μL of the obtained GO dispersion was cast on the surface of pretreated GCE and dried in air to get GO modified GCE (denoted as GO/GCE). Finally, AuPt bimetallic nanoparticles-GR nanocomposite modified GCE were obtained by immersing GO/GCE into 0.1 M PBS (pH = 5.0) containing HAuCl_4_ and H_2_PtCl_6_ with different HAuCl_4_/H_2_PtCl_6_ concentration ratios (the total concentration of HAuCl_4_ and H_2_PtCl_6_ was kept at 0.6 mM) and applying a constant potential of −1.3 V for optimal 500 s. Before deposition, the mixture solution was deaerated by purging high-purity nitrogen, and the nitrogen environment was then kept over the solution to prevent oxygen from reaching the solution. The obtained AuPt bimetallic nanoparticles-GR nanocomposite modified GCEs were denoted as Au1Pt2NPs-GR/GCE, Au1Pt1NPs-GR/GCE and Au2Pt1NPs-GR/GCE, corresponding to the HAuCl_4_/H_2_PtCl_6_ concentration ratio of 1:2, 1:1 and 2:1, respectively. For comparision, the AuNPs-GR/GCE and PtNPs-GR/GCE were prepared under similar conditions.

## 3. Results and Discussion

### 3.1. Morphological and Structural Analysis

**Figure 1:**
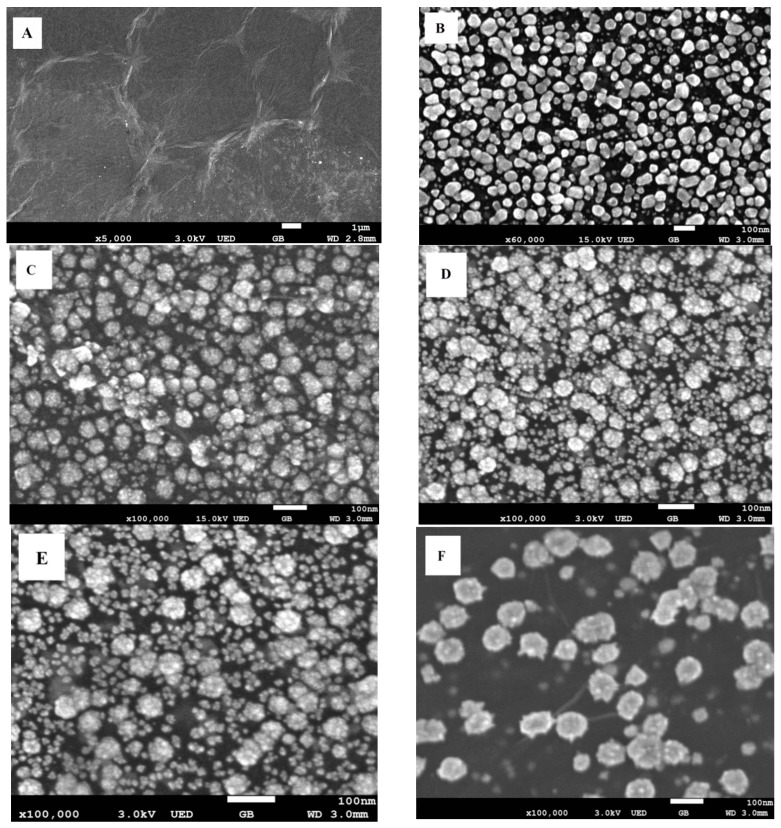

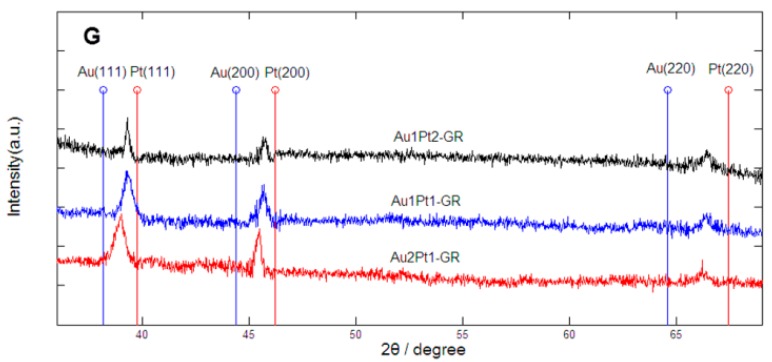
(**A**) SEM image of GR; (**B**) SEM image of AuNPs-GR; (**C**) SEM image of Au1Pt2NPs-GR; (**D**) SEM image of Au1Pt1NPs-GR; (**E**) SEM image of Au2Pt1NPs-GR; (**F**) SEM image of PtNPs-GR; (**G**) XRD patterns of Au1Pt2-GR, Au1Pt1-GR and Au2Pt1-GR.

The surface morphologies of the as-prepared nanocomposites were examined by scanning electron microscopy (SEM). As shown in Figure 1A, the surface morphology of GR film exhibited a typical wrinkled and crumpled structure. The surface morphology of AuNPs-GR (Figure 1B) showed that AuNPs with a quasi-spherical shape and relatively smooth surface were densely and uniformly distributed on the surface of GR. However, the surface morphology of PtNPs-GR (Figure 1F) showed that PtNPs with a spherical and flowerlike shape were sparsely distributed on the surface of GR sheets. It was obvious that the density of PtNPs was much lower than that of AuNPs, which indicated that the depositon of AuNPs on the surface of GR was much easier than that of PtNPs under the same electrochemical co-reduction conditions. Figure 1C–E displayed the SEM images of Au1Pt2NPs-GR, Au1Pt1NPs-GR and Au2Pt1NPs-GR, respectively. All AuPt alloy nanoparticles were formed as cauliflower-like shapes with rough surfaces, and the shapes and sizes changed with the different HAuCl_4_/H_2_PtCl_6_ concentration ratios. Specifically, with the increase of HAuCl_4_/H_2_PtCl_6_ concentration ratio (1:2, 1:1, 2:1), more smaller AuPt alloy nanoparticles appeared with an average diameter of 15 nm among the bigger cauliflower-like alloy nanoparticles with an average diameter of 45 nm. Although there were no higher magnification SEM images of the smaller AuPt alloy nanoparticles, it was assumed that these smaller AuPt alloy nanoparticles also possessed cauliflower-like shapes. The possible formation mechanisms of these cauliflower-like AuPt alloy nanoparticles was described as below: the growth of AuNPs was easier and faster on the surface of GR than that of PtNPs, and AuNPs tended to form nuclei during the growth process of AuNP bimetallic nanoparticles; subsequent growth of PtNPs occurred predominantly at the remaining Au seeds rather than onto the surface of GR; when the HAuCl_4_/H_2_PtCl_6_ concentration ratio increased, more gold seeds appeared on the surface of GR films, but there was not enough PtNPs to grow onto as-deposited gold nuclei, which resulted in more smaller AuPt alloy nanoparticles with the increase of HAuCl_4_/H_2_PtCl_6_ concentration ratio [43,44].

In order to further determine the feature of the obtained nanocomposites, an XRD study was conducted. Figure 1G showed the XRD patterns of the obtained AuPt bimetallic nanoparticles-GR nanocomposites. It was obvious that there appeared three well-resolved diffraction peaks for all three types of nanocomposites, which could be assigned to the (111), (200) and (220) planes of AuPt bimetallic nanoparticles, respectively, demonstrating a face-centered cubic (fcc) structure. As is known, the peaks of pure gold nanoparticles (2θ = 38.2°, 44.4° and 64.6°) and pure platinum nanoparticles (2θ = 39.8°, 46.2° and 67.5°) are assigned to the (111), (200), and (220) planes, respectively. As shown in Figure 1G, the (111) peaks of Au1Pt2NPs-GR, Au1Pt1NPs-GR and Au2Pt1NPs-GR samples fall well between (111) peaks of pure Au and Pt nanoparticles, suggesting that a single-phase alloy of PtAu rather than two separated phases of Pt and Au has been formed by electrochemical co-reduction.

### 3.2. Electrochemical Characterization

**Figure 2:**
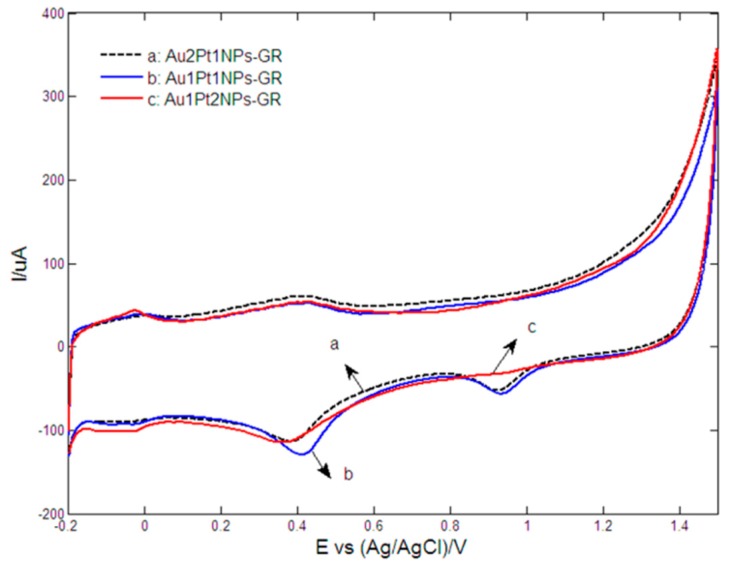
Cyclic voltammograms of (**a**) Au2Pt1NPs-GR; (**b**) Au1Pt1NPs-GR and (**c**) Au1Pt2NPs-GR nanocomposites modified electrodes in 0.5 M H_2_SO_4_ at scan rates of 100 mV/s.

Figure 2 showed the cyclic voltammetric responses of Au2Pt1NPs-GR/GCE (curve a), Au1Pt1NPs-GR/GCE (curve b) and Au1Pt2NPs-GR/GCE (curve c) in 0.5 M H_2_SO_4_. The potential peaks from −200 mV to 100 mV resulted from the hydrogen adsorption/desorption reactions. The reduction peaks at about 400 mV and 900 mV corresponded to the reduction of Au and Pt oxide species, respectively [45]. Particularly, for Au1Pt2NPs-GR/GCE, the near absence of the Au oxide reduction peak showed that Au nanoparticles were covered by Pt and few exposed Au sites still remained. However, Au1Pt1NPs-GR/GCE showed almost the largest reduction peaks of Au and Pt oxide species in H_2_SO_4_ solution, which indicated that the modified electrode possessed the highest metal electrochemical catalytic area.

**Figure 3:**
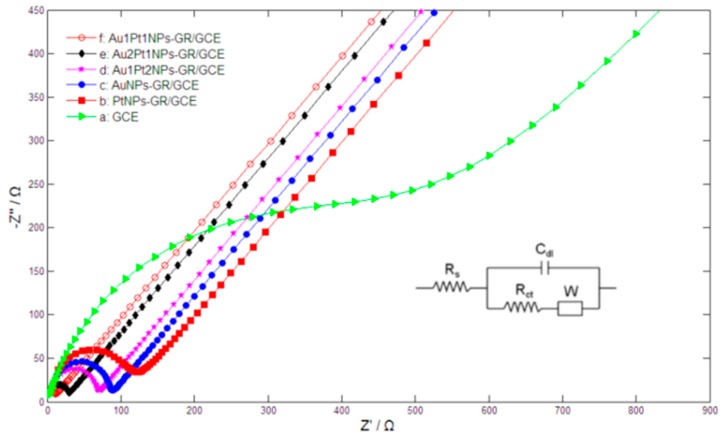
Nyquist plots of bare GCE (**a**); PtNPs-GR/GCE (**b**); AuNPs-GR/GCE (**c**); Au1Pt2NPs-GR/GCE (**d**); Au2Pt1NPs-GR/GCE (**e**) and Au1Pt1NPs-GR (**f**) in 2 mM [Fe(CN)_6_]^3−/4−^ + 0.1 M KCl solution with the frequencies swept from 10^6^ to 0.01 Hz and the AC voltage amplitude of 5 mV. Inset figure is the Randles circuit model.

The electrochemical behaviors of the different nanocomposites were further studied by electrochemical impedance spectroscopy (EIS). Figure 3 showed the typical Nyquist plots of the different electrodes in 0.1 M KCl solution containing 2 mM [Fe(CN)_6_]^3−/4−^. For bare GCE (curve a), the electron-transfer resistance (*R_ct_*) was estimated to be 496 Ω. The *R_ct_* of PtNPs-GR/GCE (curve b) and AuNPs-GR/GCE (curve c) further decreased, corresponding to 110 Ω and 79.6 Ω, respectively, which indicated that monometallic nanoparticles-GR nanocomposites could greatly enhance electron transfer rates. By comparison, the *R_ct_* of Au1Pt2NPs-GR/GCE (curve d), Au2Pt1NPs-GR/GCE (curve e) and Au1Pt1NPs-GR/GCE (curve f) obviously reduced to 60.9 Ω, 24.1 Ω and 13.9 Ω, respectively, which implied that AuPt bimetallic nanoparticles-GR nanocomposites provided more efficient electron transfer channels, faster electron transfer rates, and better electric conductivity compared with the corresponding monometallic nanoparticles-GR nanocomposites. However, of these AuPt bimetallic nanoparticles-GR nanocomposites, Au1Pt1NPs-GR/GCE possessed the smallest *R_ct_*, which illustrated that the modified electrode owned the fastest electron transfer rates and best conductivity.

### 3.3. Electrocatalytic Activity toward DA on Different Electrodes

**Figure 4:**
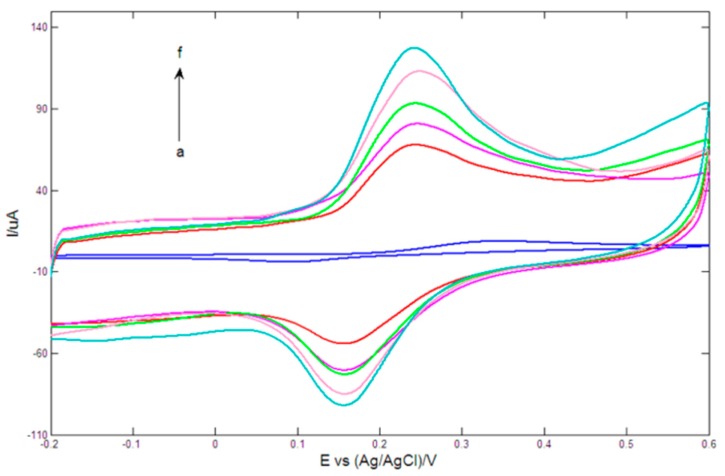
Cyclic voltammetrys of bare GCE (**a**); PtNPs-GR/GCE (**b**); AuNPs-GR/GCE (**c**); Au1Pt2NPs-GR/GCE (**d**); Au2Pt1NPs-GR/GCE (**e**) and Au1Pt1NPs-GR/GCE (**f**) in 0.1 M PBS (pH = 7.0) containing 0.2 mM DA. Scan rate: 100 mV/s.

Figure 4 showed the cyclic voltammetric responses of different electrodes in 0.2 mM DA solution prepared with 0.1 M PBS (pH = 7.0). On bare GCE (curve a), a pair of poor redox peaks with a big peak potential separation of 255 mV appeared. After modified with PtNPs-GR nanocomposite (curve b), the current responses evidently increased, and the potential difference between anodic peak potential and corresponding cathodic peak potential was remarkably reduced to 93 mV, which demonstrated that the synergistic effects of GR and PtNPs could significantly promote electron transfer rates. For AuNPs-GR/GCE (curve c), the redox peak currents further increased. However, the redox current responses of Au1Pt2NPs-GR/GCE (curve d), Au2Pt1NPs-GR/GCE (curve e) and Au1Pt1NPs-GR/GCE (curve f) were obviously bigger than that of PtNPs-GR/GCE and AuNPs-GR/GCE, which indicated that AuPt bimetallic nanoparticles-GR nanocomposites possessed faster electron transfer rates and better electrocatalytic activity toward DA compared with the corresponding monometallic nanoparticles-GR nanocomposites. Of these three bimetallic nanoparticles-GR nanocomposite modified GCE, Au1Pt1NPs-GR/GCE displayed the biggest redox current responses and lowest peak potential difference (only 85 mV), which was consistent with the results of EIS. The above results implied that when the HAuCl_4_/H_2_PtCl_6_ concentration ratio was 1:1 during the preparation of bimetallic nanoparticles-GR nanocomposites, the obtained electrode (Au1Pt1NPs-GR/GCE) showed the best electrocatalytic activity toward DA. Therefore, Au1Pt1NPs-GR/GCE was used to selectively detect DA in the following experiments.

### 3.4. Effect of pH Value on the Oxidation of DA

**Figure 5:**
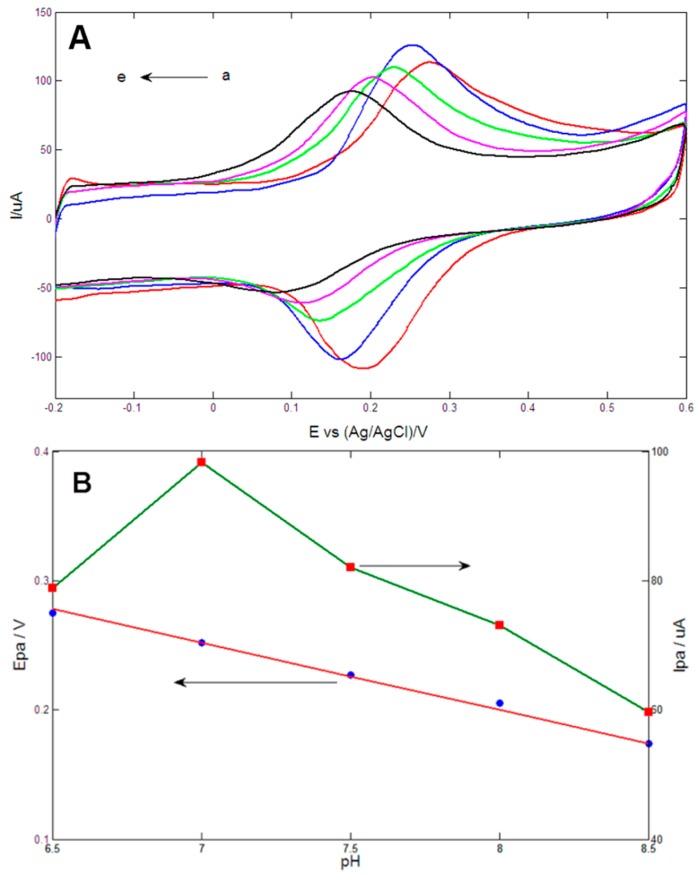
(**A**) Cyclic voltammetrys of Au1Pt1NPs-GR/GCE in 0.1 M PBS (pH values from *a* to *e*: 6.5, 7, 7.5, 8.0, 8.5) containing 0.2 mM DA at scan rate of 100 mV/s; (**B**) Plots of anodic peak potential (*E_pa_*) and anodic peak current (*I_pa_*) *vs.* pH value.

The above results showed that Au1Pt1NPs-GR/GCE displayed the best electrocatalytic activity toward DA and thus was used to selectively detect DA. In this section, the effects of pH value on DA oxidation at the Au1Pt1NPs-GR/GCE were systematically studied. Figure 5A showed cyclic voltammetric responses of the obtained electrode in 0.1 M PBS (pH values from *a* to *e*: 6.5, 7, 7.5, 8.0, 8.5) containing 0.2 mM DA. It was obvious that the anodic peak current (*I_pa_*) reached the maximum value at pH = 7.0, and therefore, pH 7.0 was selected to detect DA in the following experiments. As shown in Figure 5B, anodic peak potential (*E_pa_*) linearly decreased as pH changed from 6.5 to 8.0, and the linear regression equation could be expressed as *E_pa_* (V) = 0.616 − 0.052 pH (*R^2^* = 0.991). The slope of −52 mV/pH was close to the theoretical value of −59 mV/pH calculated by the Nernst equation, which indicated the same number of electrons and protons involved in DA oxidation reaction at the Au1Pt1NPs-GR/GCE [46].

### 3.5. Effects of Scan Rate on the Oxidation of DA

**Figure 6:**
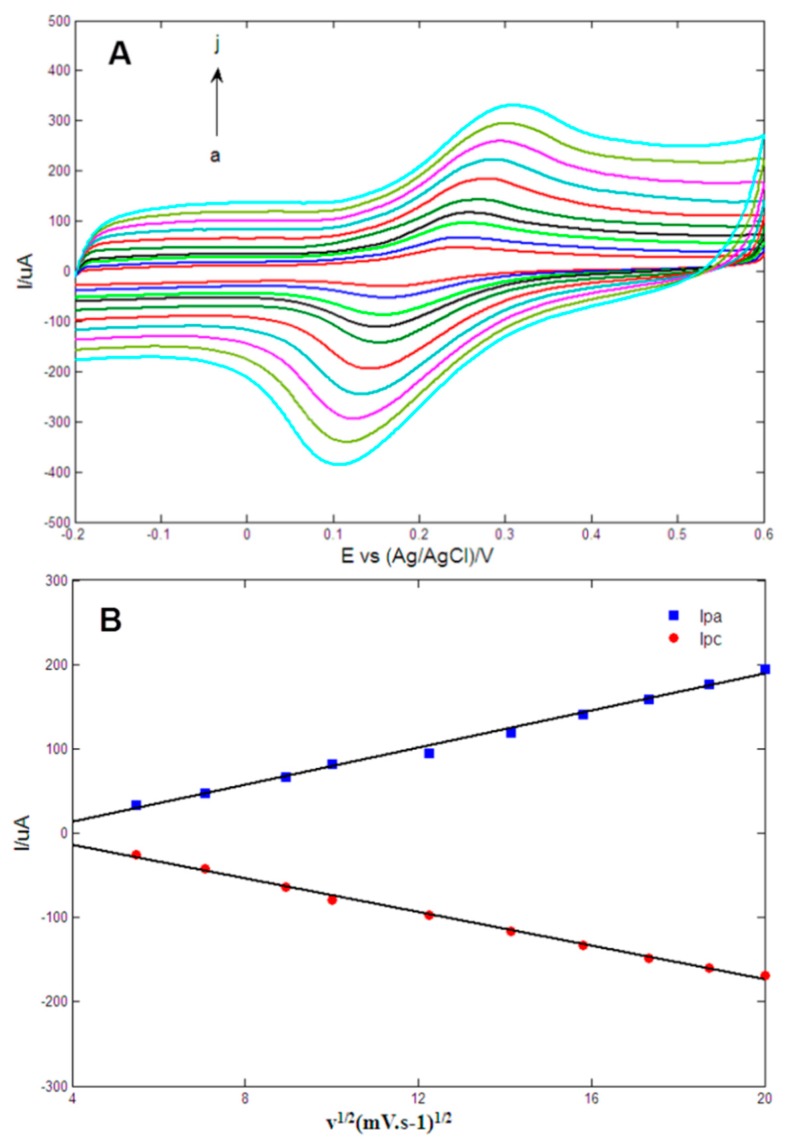
(**A**) Cyclic voltammetrys of Au1Pt1NPs-GR/GCE in 0.1 M PBS (pH = 7.0) containing 0.2 mM dopamine at 30, 50, 80, 100, 150, 200, 250, 300, 350 and 400 mV/s (from *a* to *j*); (**B**) Plots of anodic (*I_pa_*) and cathodic peak currents (*I_pc_*) *vs.* scan rates.

The effects of scan rate on the electrochemical oxidation at the Au1Pt1NPs-GR/GCE was studied using cyclic voltammetry (CV) techniques in 0.1 M PBS (pH = 7.0) containing 0.2 mM DA with the scan rates varying from 30 mV/s to 400 mV/s. As shown in Figure 6A, as the scan rate increased, the anodic peak potentials (*E_pa_*) became more positive whereas the cathodic peak potentials (*E_pc_*) became more negative, which indicated that the charge transfer rate slowed and that the reversibility of electrochemical redox reaction of DA became poor. Figure 6B implied that the anodic (*I_pa_*) and cathodic (*I_pc_*) peak currents displayed a linear relationship with the square root of scan rates, and the linear regression equations of *I_pa_* (μA) = −31.013 + 10.993*v*^1/2^ (mV/s)^1/2^ and *I_pc_* (μA) = 25.761 − 9.9627*v*^1/2^ (mV/s)^1/2^ with respective correlation coefficients of *R^2^* = 0.9926 and 0.9964 were obtained, which indicated that the electrochemical oxidation of DA at the obtained electrode was a typical quasi-reversible diffusion-controlled process [39].

For the diffusion-controlled process, the relation between *I_pa_* (uA) and the diffusion coefficient of electroactive species, D_o_ (cm^2^·s^−1^), was expressed as the following equation:

$$I_{pa}=\left(2.69\times 10^{5}\right)n^{3/2}{\text{AC}}_{0}^{\text{*}}D_{o}^{1/2}v^{1/2}$$

where *n* is the electron number involved in oxidation reaction, A is the geometrical electrode area, *C_0_* is the analyte concentration and *v* is the scan rate (V/s). Here, *n* is 2, A is 0.0706 cm^2^ and *C_0_* is equal to 2 × 10^−4^ mol/cm^3^. According to the linear relationship between *I_pa_* and *v*^1/2^ (*I_pa_* (μA) = 30.6415 + 426.851*v*^1/2^ (V/s)^1/2^, *R^2^* = 0.996), the diffusion coefficient of the oxidation process was computed to be 1.01 × 10^−7^ cm^2^/s, which was in agreement with the value of 2.4 × 10^−7^ cm^2^/s reported in the literature [47] but smaller than the value of 5.8 × 10^−^^6^ cm^2^/s reported in the literature [48].

In addition, the *E_pa_* and *E_pc_* were proportional to the values of log *v*, and linear regression equations of *E_pa_*(V) = 0.0594log *v* (V/s) + 0.3237 (*R^2^* = 0.9941) and *E_pc_*(V) = −0.0497log *v* (V/s) + 0.0996 (*R^2^* = 0.9938) were obtained. The slope of the linear relationship between *E_pa_* and log *v* can be expressed as 2.3*RT/(1 −* α*)nF*, where *R*, *T* and *F* are constants, *n* is the electron number, and α is the coefficient of electron transfer [43]. Therefore, the electron number *n* was computed to be 1.82 ≈ 2 assuming α = 0.5 for a quasi-reversible process.

### 3.6. Selective Detection of DA

**Figure 7:**
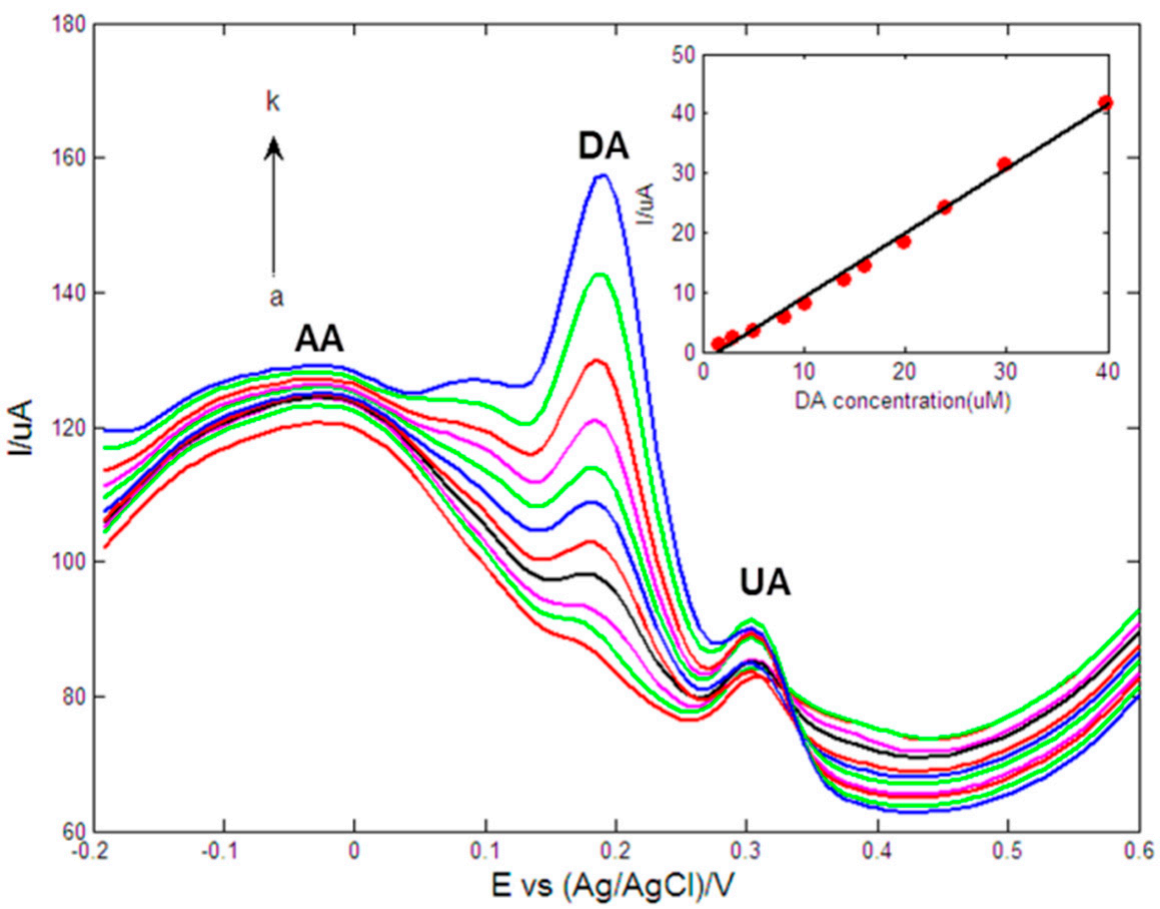
DPVs of Au1Pt1NPs-GR/GCE in 0.1 M PBS (pH = 7.0) containing 50 μM AA, 12 μM UA and different concentrations of DA (from *a* to *k*: 1.6, 3, 5, 8, 10, 14, 15.9, 19.9, 23.9, 29.8 and 39.7 μM). Inset: The calibration curve of DA. Scan rate: 10 mV/s, pulse amplitude: 50 mV, pulse width: 40 ms, pulse period: 0.3 s.

Figure 7 showed the DPV responses of the obtained electrode in 0.1 M PBS (pH = 7.0) containing different concentrations of DA in the presence of 50 μM AA and 12 μM UA at the Au1Pt1NPs-GR/GCE. It could be observed that three peak potentials of AA, DA and UA appeared at −0.07 V, 0.17 V and 0.30 V, respectively. In addition, the potential separations of the DPV oxidation peak were estimated to be 177 mV, 130 mV and 307 mV for DA and AA, DA and UA, and AA and UA, respectively. The separations were large enough to allow the selective detection of DA in the presence of AA and UA. Results implied that the DPV peak currents were proportional to DA concentrations in the range of 1.6–39.7 μM and a linear regression equation of *I_pa_* (μA) = −1.7914 + 1.0806*C* (μM) (*R^2^* = 0.9943). The limit of detection (LOD) was computed to be 0.1 μM (*S/N* = 3), based on the following equation where σ is the standard deviation of five measurements and *S* is the slope of the calibration line:

$$\text{LOD}=\frac{3\text{σ}}{S}$$

The comparison of detection characteristics of the proposed modified electrode with other electrodes used for selectively detecting DA was illustrated in Table 1. It was obvious that the linear range, detection limit and sensitivity obtained in this work were comparable to or better than some previous reports. Compared to the literature data in Table 1, the biggest advantage of the sensor in this work might be a high sensitivity, and the biggest drawback might be a slighty narrow linear range.

**Table 1:** Comparison of detection performance for dopamine with other electrodes reported in the literatures.

Electrode	Detection Method	Sensitivity (μA/μM)	Linear Range (μM)	Detection Limit (μM)	References
PtAu hybrid film/GCE	DPV	0.05	24–384	24	[1]
Poly-CDDA/GCE	DPV	0.054	5–280	0.29	[49]
Poly (Evans Blue)/GCE	DPV	0.33	1–10	0.25	[50]
Poly (PEDOT-PANS)/GCE	LSV	1	2–10	0.5	[51]
ZnO/RM/GCE	CV	0.25	6–960	0.7	[52]
Pt/RGO/GCE	DPV	0.0391	10–170	0.25	[53]
Chitosan-Graphene/GCE	DPV	-	1–24	1	[54]
Sol–gel carbon composite electrode	SWV	0.7	0.5–20	0.1	[55]
polyaniline/polypyrrole nanofibre-graphene modified electrode	SWV	0.866	0.0001–100	0.00005	[56]
3D-RGO/GCE	CA	244.17	5–1000	0.17	[57]
AuCo alloy nanoparticles-Graphene/GCE	DPV	0.8639	2.1–21.1	0.1	[58]
Au1Pt1NPs-GR/GCE	DPV	1.0806	1.6–39.7	0.1	This work

Note: GCE stands for glassy carbon electrode; DPV stands for differential pulse voltammetry; CDDA stands for 3-(5-chloro-2-hydroxyphenylazo)-4,5-dihydroxynaphthalene-2,7-disulfonic acid; PEDOT-PANS stands for 3,4-ethylenedioxythiophene-co-(5-amino-2-naphthalenesulfonic acid); LSV stands for linear sweep voltammetry; ZnO/RM stands for zinc oxide/redox mediator; CV stands for cyclic voltammetry; RGO stands for reduced graphene oxide; SWV stands for square wave voltammogram; CA stands for chronoamperometry.

### 3.7. Reproducibility and Stability

The fabrication reproducibility, storage stability and operational stability of Au1Pt1NPs-GR/GCE were studied by detecting DPV peak currents of DA in 0.1 M PBS (pH = 7.0) containing 10 μM DA in the presence of 50 μM AA and 12 μM UA. Fabrication reproducibility was examined by using ten different proposed modified electrodes prepared under the same conditions, and a relative standard deviation (RSD) of 4.2% was obtained. Storage stability was evaluated by measuring DPV peak currents once a day over a period of two weeks, and the electrodes were stored dry at 4 °C in the refrigerator when not used. The peak current remained at 92% of the initial current after one week and gradually decreased to 89% after two weeks. Operational stability was studied by 10 sequential determinations of 10 μM DA every 10 min, and a RSD of 3.7% was obtained. These results revealed the excellent reproducibility and stability of the prepared electrode.

### 3.8. Interference Study

In addition to AA and UA, the influences of other organic and inorganic species that coexisted with DA were also examined. The RSD (*n* = 5) of 600-fold sodium chloride, magnesium sulfate, potassium chloride, 200-fold glucose, and 100-fold cysteine, lysine, citric acide, aspartic acid for the detection of 10 μM DA in the presence of 50 μM AA and 12 μM UA was 1.6%, 1.9%, 1.7%, 3.4%, 3.1%, 2.8%, 3.2% and 3.6%, respectively, which indicated that these interferons had no significant influences on the detection of DA and the obtained electrode displayed excellent selectivity toward DA.

### 3.9. Real Sample Analysis

To investigate the application potential of the obtained electrodes for the detection of DA, the recovery tests were carried out in human urine samples. Human urine samples were collected from healthy laboratory volunteers, and were diluted three times using 0.1 M PBS (pH = 7.0). Results of recovery tests were shown in Table 2. Positive results showed that the proposed method was simple and convenient enough to be used to detect DA in urine samples.

**Table 2:** Results of recovery tests for the detection of DA in human urine samples.

No.	Added (μM)	Detected (μM)	Recovery (%)
1	5	5.2	104
2	10	10.1	101
3	15	14.9	99.3
4	20	19.8	99
5	25	25.3	101.2

## 4. Conclusions

In this paper, AuPt bimetallic nanoparticles-graphene nanocomposites were prepared by electrochemical co-reduction of graphene oxide, HAuCl_4_ and H_2_PtCl_6_. The obtained Au1Pt1NPs-GR/GCE showed excellent sensitivity and selectivity toward DA and was used to detect DA in the presence of AA and UA with a wide linear range and a low detection limit. The prepared electrode was also used for detection of DA in real samples and satisfactory results were obtained.
